# Formulated Beta-Cyfluthrin Shows Wide Divergence in Toxicity among Bird Species

**DOI:** 10.1155/2011/803451

**Published:** 2011-03-17

**Authors:** Laura M. Addy-Orduna, María-Elena Zaccagnini, Sonia B. Canavelli, Pierre Mineau

**Affiliations:** ^1^Estación Experimental Agropecuaria Paraná, Instituto Nacional de Tecnología Agropecuaria (INTA), Ruta 11 km 12.5, Paraná, 3100 Entre Ríos, Argentina; ^2^Instituto de Recursos Biológicos, Instituto Nacional de Tecnología Agropecuaria (INTA), Castelar, 1712 Buenos Aires, Argentina; ^3^Science and Technology Branch, Environment Canada, Ottawa, ON, Canada KIA 0H3

## Abstract

It is generally assumed that the toxicity of pyrethroid insecticides to birds is negligible, though few species have been tested. The oral acute toxicity of formulated beta-cyfluthrin was determined for canaries (*Serinus sp.*), shiny cowbirds (*Molothrus bonariensis*), and eared doves (*Zenaida auriculata*). Single doses were administered to adults by gavage. Approximate lethal doses 50 (LD_50_) and their confidence intervals were determined by approximate D-optimal design. Canaries were found to be substantially more sensitive to formulated beta-cyfluthrin (LD_50_ = (170 ± 41) mg/kg) than the other two species tested (LD_50_ = (2234 ± 544) mg/kg and LD_50_ = (2271 ± 433) mg/kg, resp.). The LD_50_ obtained for canaries was also considerably lower than typical toxicity values available in the literature for pyrethroids. This study emphasizes the need for testing a broader range of species with potentially toxic insecticides, using modern up and down test designs with minimal numbers of birds.

## 1. Introduction

The widespread use of pesticides contributes to population declines and mortality of birds in agroecosystems [1]. Among the various categories of pesticides, insecticides typically present a higher risk of acute effects [2, 3] because of their elevated inherent toxicities and high potential for exposure. Documented cases of mass mortality by intoxication and the various studies reporting negative effects of insecticides on birds are clear evidence of the risk posed by insecticides on wild bird species (e.g., [2–11]). One case of mass mortality of birds that received a great deal of attention in Argentina was the 1995-1996 mortality of Swainson's hawks caused by monocrotophos, an organophosphorus insecticide [12, 13]. After this event, monocrotophos registration was cancelled in Argentina while the pyrethroid insecticides gained in importance and popularity. 

Among insecticides, pyrethroids are a class of neurotoxic synthetic insecticides widely used due to their relative safety to mammals and birds, high insecticidal potency at low dosages, and fast biodegradation [14]. Insect axon sodium channels are 100-fold more sensitive to pyrethroid esters than mammalian channels [15]. For these reasons, pyrethroids have gradually replaced organochlorine, organophosphate, and carbamate insecticides in the field. Several studies about pyrethroids have been conducted on vertebrates (e.g., [16–18]), the majority on rodents (e.g., [19–23]). The neurotoxicity of pyrethroids to mammals depends on the stereochemical configuration and the pyrethroid structure [24, 25]. In contrast, little is known of the toxicity of pyrethroids to birds, probably because this class of insecticides is generally considered to have negligible toxicity to birds. 

Beta-cyfluthrin (cyano-(4-fluoro-3-phenoxyphenil)-methyl-3-(2,2-dichloroethenyl)-2,2-dimethyl-cyclopropanecarboxylate) is the active ingredient of insecticide formulations used to control a wide variety of pests on cotton, corn, sunflower and soybean crops. Like other pyrethroids, beta-cyfluthrin presents stereoselective interaction with a fraction of the sodium channels of the neuronal membranes, resulting in a prolongation of the inward sodium currents evoked in neurons by every incoming pulse of excitatory stimulation [26–28]. Beta-cyfluthrin is a mixture of four diastereomers, with diastereomers II and IV predominating and determining chemical and physical properties of the substance [29, 30]. Beta-cyfluthrin is a type II pyrethroid, with a characteristic cyano group on the alpha carbon. Type II pyrethroids present greater insecticidal effectiveness and higher toxicity than type I pyrethroids. Type II esters keep the sodium channel open for a more prolonged time period than type I esters [31]. The main signs of intoxication of type II pyrethroids in mammals include choreoathetosis and salivation (CS) [25]. 

The reported LD_50_ of beta-cyfluthrin to birds in the Pesticide Manual [32] is >2000 mg/kg in the Japanese quail. The USEPA in its “ECOTOX” database (http://cfpub.epa.gov/ecotox/) gives the same value, but associated with the northern bobwhite. Yet, unpublished information cited in a report without details about vehicle or formulation gave an LD_50_ of ca. 100 mg/kg for beta-cyfluthrin in canaries as well as the more usual values for northern bobwhite and mallard duck of >2000 mg/kg [29]. This value for canaries, if real, casts doubts on the general wisdom that pyrethroids are non-toxic to birds. The present study had for objective independent corroboration of the LD_50_ of beta-cyfluthrin to canaries and determining the acute oral toxicity of commercially formulated beta-cyfluthrin to two novel and wild species, the shiny cowbird (*Molothrus bonariensis*) and the eared dove (*Zenaida auriculata*).

## 2. Materials and Methods

### 2.1. Site and General Conditions of Study

The study was carried out in the research facilities of the INTA (Instituto Nacional de Tecnología Agropecuaria) at the Paraná Agricultural Experimental Station (31°50′53′′S, 60°32′19′′W). The study was carried out in an aviary of 20 × 10 m, including an acclimation area with 6 groups of pens (each 3 × 2 × 3 m) and 24 individual test cages (each 0.5 × 0.5 × 0.5 m). The photoperiod and the average temperature of the testing room during the dosing were recorded (Tables 2 and 3). The ventilation was controlled so as to maintain the indoor conditions of temperature and humidity within outdoor environmental ranges.

### 2.2. Selection, Capture, and Housing of Birds

The wild birds, shiny cowbirds, and eared doves were selected based on their large numbers in surrounding fields, which assured their availability, abundance, and capture success. Shiny cowbirds were captured with mist-nets and eared doves with bait traps. Captive bred canaries were used. Healthy adult birds were weighted and grouped by sex before being acclimated to experimental conditions for at least 14 days. At least three 1.5 m-perches were placed in each pen. Shiny cowbirds were fed insectivore certified commercial food, eared doves were offered a mix of wheat and sunflower seeds, and canaries, a commercial seed mix and ground egg. Bottled water for human consumption was offered *ad libitum *to all species. Because of the absence of a constituted animal care committee at INTA or at the local university (Universidad Nacional del Litoral) which provided academic supervision of this research, guidelines of the Denver Wildlife Research Center of the US Department of Agriculture were followed for the capturing, transportation, housing, care, euthanasia, and necropsy of the birds, in addition to other procedures of the study [33–45].

### 2.3. Chemical and Dose

To obtain the test doses (mg beta-cyfluthrin/kg body weight), we used a commercial formulation (Bulldock of Bayer CropScience), a suspension of 12.5 g a.i./100 mL of unreported inert ingredients. We assumed label concentration was correctly reported and administered to birds the necessary volume of formulated product corresponding to the required dose of beta-cyfluthrin. It is known that pyrethroid toxicity may be greatly influenced by the dosing vehicle [46]. Because wild birds are exposed to formulated products, we opted to test the formulation without an additional vehicle where possible and with distilled water as a diluent for several doses for canaries (see dilutions in the footnotes of Table 3). 

Doses were calculated according to standard equations for each stage of the approximate D-optimal design [47], in milligrams of a.i. per kilogram of body weight, as shown in Table 3. The dosing volumes were calculated based on individual body weights measured within 12 hours of dosing (Table 1). To prevent regurgitation, the higher dose volumes (>0.17 mL for canaries, >0.45 mL for shiny cowbirds, and >1.0 mL for eared doves) were split and administered in up to four aliquots separated by 15 minutes. This split administration of doses took place for all species in the limit test, one canary in the first stage of the full test and all shiny cowbirds and eared doves in all stages of the full test (Table 1). Dose volumes never exceeded 16 mL/kg BW (body weight) in canaries, 27 mL/kg BW in shiny cowbirds, and 26 mL/kg BW in eared doves. The formulated test chemical was given by gavage. The catheter was lubricated with Vaseline to diminish possible discomfort when introduced. Individuals that regurgitated part or all of a dose and who survived the dose were substituted for others due to the fact that regurgitation modifies the dose and prevents the correct approximation of the LD_50_ [48]. Forty-six percent of shiny cowbirds, 33% of eared doves and 16% of canaries regurgitated, despite being fasted before the dose.

### 2.4. Procedure

Acute oral toxicity tests were carried out following draft Guideline 223 of the Organisation for Economic Cooperation and Development [47]. This procedure minimizes the number of birds used and has extensive statistical validation. 

**Figure 1:**
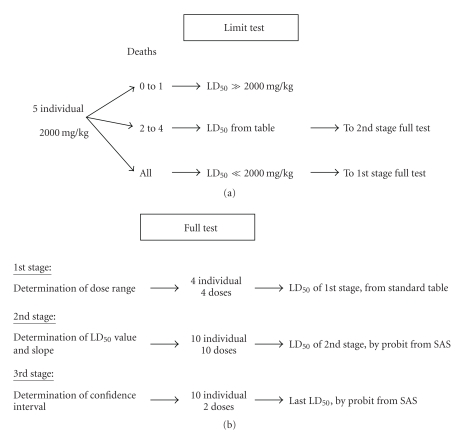
Diagram of methodology used.

First, five individuals of each species were treated with a limit dose of 2000 mg/kg of test chemical. Following any mortality at this limit dose, LD_50_ were estimated in sequential stages with the approximate D-optimal design (full test; Figure 1). In canaries, the first stage of the full test was carried out to confirm and improve the initial estimate of the canary LD_50_ (250 mg/kg, based on the aforementioned literature value and the result of a limit test). An additional stage was added to obtain a greater level of precision.

Birds were randomly assigned to each test and were observed for 14 days after the dose. Mortality, clinical symptoms, change in weight between the beginning and the end of the study, regurgitations, time to death (in hours), and recovery were recorded.

Both test and control animals were examined by necropsy to determine macroscopic differences. The size, position and appearance of all organs and the full g.i. tract were examined. Also, livers and hearts were weighed and their relative weights calculated (1), in order to detect any pathology associated with any loss or increase in mass of these organs (hepatomegaly, necrosis, hypertrophy, etc.).

(1) $$\begin{array}{c} R_{L}=\left(\frac{\text{liver}\;\text{weight}}{\text{body}\;\text{weight}}\right)*100,\\ R_{H}=\left(\frac{\text{heart}\;\text{weight}}{\text{body}\;\text{weight}}\right)*100. \end{array}$$

### 2.5. Statistical Analysis

We fit a probit model [49] to the combined data from all stages (STAT-SAS 6.1) to obtain the LD_50_ estimates, confidence intervals confidences and slopes of dose-response curves. Both the initial and final body weights and the relative weights of hearts and livers were compared by one-way ANOVA using SPSS v.10 for Windows.

## 3. Results

### 3.1. Limit Tests

Initial LD_50_ estimates obtained for the limit tests were 2247 mg/kg for both shiny cowbirds and eared doves because 40% of individuals died in both species. By contrast, all treated canaries died, and it was, therefore, impossible to obtain an initial estimate of LD_50_ with the limit test (Table 2). 

### 3.2. Full Test

**Figure 2:**
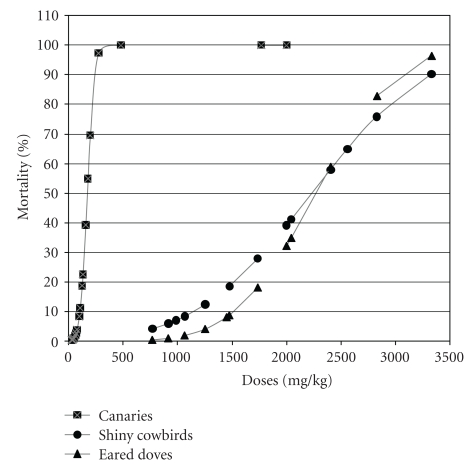
Dose-response curves.

With canaries, the LD_50_ values estimated at each sequential stage were 68 mg/kg, 110 mg/kg and 170 mg/kg, respectively. During the additional stage (similar to the third stage, performed in order to decrease the confidence intervals of the LD_50_), two of four individuals that received the highest dose regurgitated, and, for this reason, they were not included in the results. For shiny cowbirds and eared doves, although the doses administered in the second stage were the same because of similar results in the limit test, the mortality was different (Table 3). The LD_50_ estimates after the second stage were 1589 mg/kg and 2338.6 mg/kg for shiny cowbirds and eared doves, respectively. The final LD_50_ estimates, obtained by fitting a probit model to the combined data of all stages for each species were 170 ± 41 mg/kg for canaries, 2234 ± 544 mg/kg for shiny cowbirds, and 2271 ± 433 mg/kg for eared doves. The dose-response curves are shown in Figure 2.

**Figure 3:**
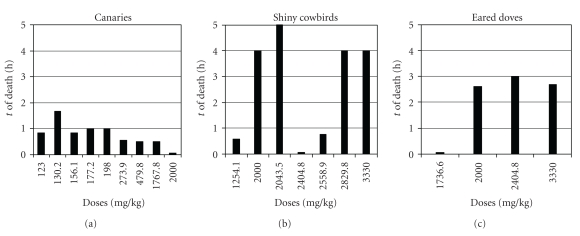
Time of death as a function of dose (only the doses that caused death are shown).

Clinical signs included ruffled appearance, salivation (evidenced by constant deglutition movements and head shaking), decreased activity, prostration, panting, labored breathing, body tremor, balance loss and/or convulsions. Signs appeared shortly after dosing and lasted from a few minutes to a few hours. There were doses that did not produce clinical signs and others that allowed recuperation of individuals with signs of intoxication, including convulsions (Tables 2 and 3). All recuperations were within the first 24 hours after the dosage. Predose and 14-day postdose weights are given in Table 4. There were no significant differences between the body weights of the survivors before dosing and 14 days after the dose, except for canaries in the third stage of the full test where 14-day postdose weights were higher than predose weights (*P* = .018). Maximum time to death was 1.75 hours in canaries, 3 hours in eared doves, and 5 hours in shiny cowbirds. Only canaries showed a tendency toward a shorter time to death with increasing dose (Figure 3). 

All birds that died presented stiffness of fore and back limbs. We observed a white thick liquid in different sections of the g.i. tract, attributable to the insecticide formulation. Macroscopic differences among organs of treated and control individuals were not detected. Relative weights of heart and liver (*R*_*L*_ and *R*_*H*_) did not vary either (*P* > .05 in all cases).

## 4. Discussion

The LD_50_ value we obtained in canaries approximates the value presented in the report on beta-cyfluthrin of the European Commission [29], confirming it as moderately toxic or class II for this species. LD_50_ values for formulated beta-cyfluthrin obtained in shiny cowbirds (another passerine species) and eared doves, are close to the values reported for bobwhite quail and Japanese quail [29, 30], confirming beta-cyfluthrin to be practically non-toxic or class III for those species. Some care is needed before attributing these sensitivity differences solely to phylogeny. Variation in dosing procedures might have introduced unwanted variation in our results. These are discussed in detail below. 

The different sensitivity to beta-cyfluthrin between canaries and other tested birds may be due to differences in the characteristics of the sites of toxic action, the intestinal absorption, the metabolism, and/or the elimination of this substance. For instance, the low acute toxicity of cypermethrin to quail had been explained by a high resistance of the CNS of quails to the lethal effects of cypermethrin, a greater extensive metabolism to a large number of products, and a rapid elimination in the excreta [50]. It is possible that similar physiological mechanisms occur in shiny cowbirds and eared doves, explaining their low susceptibility to beta-cyfluthrin. In addition, both significant differences in sensitivity at the sites of toxic action and metabolic differences and different main detoxification routes and enzymatic activities [51–59] may play a major role in the differential responses to these insecticides. On the other hand, differences in body size among the three bird species included in this study could have influenced the results. Based on allometric research carried out by Mineau et al. [60], the small body size of the canary compared to the other two species might have contributed to the higher sensitivity.

Factors related to the experimental setting and our procedures could have influenced the results of this study. A relationship between temperature and the toxic effects of pyrethroids has been shown for several groups of animals, including insects, amphibians and mammals [51, 61–64]. In our study, variations were noted in the ambient temperatures at the time of each stage of testing (Table 3). However, even when variation was slightly higher in the case of shiny cowbirds, it is unlikely to have influenced results because we obtained the LD_50_ through combined data from sequential stages with different temperatures. Additionally, the varying dose volumes and split administrations may have increased variability between experimental animals [61]. In our work canaries were administered a volume well below those used for the other two species (Table 1). Wolansky et al. [65] showed that increasing the amount of corn oil delivered with a dose of bifenthrin, another synthetic pyrethroid, changed the time course and potency of the pesticide in rats (a two-fold difference in potency was seen for a 5-fold increase in corn oil). The use of oil as dosing agent should be avoided when highly lipophilic pesticides are being tested. We hope to have avoided this problem by dosing with the neat formulation where possible, and therefore not changing the relative concentration of pesticide in the dosing solution. Comparing the number of aliquots (Table 1) to the mortality in the tests (Table 3), suggests that the exact dosing regime probably did not affect our test results to any great extent. Finally, because we tested formulated material (with unknown inerts) rather than the active ingredient, we cannot ascertain definitively whether the canary is more sensitive to the active ingredient or to one of the formulants. However, the similarity to the value cited for the a.i. by the European Union and the relative lack of toxicity of the formulants in the other two species (with LD_50_ values similar to those obtained in quail or mallard with the active ingredient alone) suggests differences in sensitivity to the pyrethroid and not to the inerts included in the formulated material.

Clinical symptoms observed in response to high doses in the three species of bird are in agreement with those described by Sheets et al. [66] in rats treated with beta-cyfluthrin and those observed by Qadri et al. [67], who tested permethrin and cypermethrin in chickens. These symptoms consisted of decreased activity, tremors in the whole body, salivation, agitated breathing, flattened posture, and choreoathetosis. Nervous intoxication symptoms were observed a short time after intake and lasted up to a few hours, indicating that the removal of pyrethroids from the nervous system is rapid [68]. The survivors from all species did not show loss of body weight, at least by the end of the 14-day observation period (Table 4). Moreover, the body weights of canaries increased significantly during the third stage of the full test. Singh et al [69] observed increased body weights after acute treatment with beta-cyfluthrin on Albino rats. These authors postulate that the increase in body weight may be due to excessive food and water intake and increased food conversion efficiency of treated groups as compared to controlled ones. 

All deaths occurred within 24 hours after the dose, probably because peak levels in blood, liver, muscle and brain are reached the first day of treatment [25, 70]. Times to death in shiny cowbirds and eared doves were quite similar to those obtained with fenvalerate by Mumtaz and Menzer [71] in Japanese quail (4 to 8 hours). Rapid recovery is a characteristic of poisoning with pyrethroids in mammals [72]. In the three bird species, even individuals given doses approaching the LD_50_ recovered quickly (Table 3). Pascual et al. [48] reported a high frequency of regurgitation in doves as did we. Regurgitations were not as frequent in canaries, suggesting differences in their physiological capacity to regurgitate. Since the individuals that regurgitated were substituted for others, the results were not influenced by regurgitation in this study.

Acute and subacute studies have shown that pyrethroids at high doses cause liver hypertrophy, and, if death does not occur, these changes have been shown to be reversible [72, 73]. Nevertheless and as in Yavasoglu et al. [74], who worked with cypermethrin in rats, relative liver weights (*R*_*L*_)—as an indirect measure of changes in liver health or function—did not show any effect of dose. Although there are *in vitro* studies regarding the effects of pyrethroids on the cardiac muscle of rats and guinea pig [75–77], effects associated with loss or increase of heart mass were not detected here.

In conclusion, although there were factors that possibly exacerbated the differences of susceptibility within or between species (e.g., varying dose volumes, multiple dosing scheme, variable ambient temperatures, body size, etc.), the high sensitivity of canaries to beta-cyfluthrin was corroborated. On the other hand, formulated beta-cyfluthrin was found to be practically non-toxic to shiny cowbirds and eared doves. These results emphasize the need to test a broader range of species before generalizing about the toxicity of pyrethroids (and possibly other pesticides) to birds. In the case of beta-cyfluthrin specifically, although low application rates are generally used in the field (7.5–20 g a.i./ha according to Tomlin [32]), it is necessary to consider the potential variation in the toxicity of this pesticide in order to fully assess its safety to birds. In the present case, a species sensitivity approach [78] would suggest that other species of birds, especially small bodied species, will show higher sensitivity to pyrethroids. Future research is needed to explain why the canaries are more sensitive to beta-cyfluthrin, to determine whether canaries are similarly more sensitive to other pyrethroids, and more importantly, whether wildlife species related phylogenetically to canaries also present a high sensitivity to pyrethroids.

## Figures and Tables

**Table 1:** Dosing volumes (mL) and number of aliquots separately administered (in brackets).

Individual	1	2	3	4	5	6	7	8	9	10
Limit test										

Canaries	0.33 [2]	0.33 [2]	0.30 [2]	0.32 [2]	0.28 [2]					
Shiny cowbirds	0.85 [3]	1.00 [3]	0.75 [3]	0.80 [3]	0.99 [3]					
Eared doves	1.97 [3]	2.13 [3]	1.86 [3]	2.26 [3]	1.79 [3]					

1st stage of the full test										

Canaries	0.12 [1]	0.13 [1]	0.16 [1]	0.24 [2]						

2nd stage of the full test										

Canaries	0.08 [1]	0.08 [1]	0.12 [1]	0.16 [1]	0.10 [1]	0.13 [1]	0.14 [1]	0.09 [1]	0.11 [1]	0.15 [1]
Shiny cowbirds	0.31 [2]	0.36 [2]	0.48 [2]	0.5 [3]	0.68 [3]	0.67 [3]	0.81 [3]	0.82 [3]	1.5 [4]	1.34 [4]
Eared doves	0.81 [2]	0.83 [2]	0.87 [2]	1.35 [2]	1.46 [2]	1.39 [2]	2.34 [3]	1.94 [3]	2.20 [3]	3.09 [3]

3rd stage of full test										

Canaries	0.12 [1]	0.10 [1]	0.10 [1]	0.10 [1]	0.09 [1]	0.13 [1]	0.13 [1]	0.13 [1]	0.14 [1]	0.16 [1]
Shiny cowbirds	0.45 [2]	0.45 [2]	0.44 [2]	0.35 [2]	0.35 [2]	1.26 [3]	1.00 [3]	1.00 [3]	1.03 [3]	1.07 [3]
Eared doves	1.44 [2]	1.24 [2]	1.42 [2]	1.02 [2]	1.24 [2]	2.88 [3]	2.77 [3]	2.66 [3]	3.09 [3]	2.09 [3]

3rd stage of the full test (2)										

Canaries	0.11 [1]	0.13 [1]	0.11 [1]	0.13 [1]	0.10 [1]	0.11 [1]	0.11 [1]	0.09 [1]		

**Table 2:** Mortality with 2000 mg/kg of test substance (limit test).

Individual	1	2	3	4	5	*T* (°C)	*P*
Canaries	X	X	X	X	X	22.6	12.7
Shiny cowbirds	O^†^	O	O^†^	X	X	12.9	11.3
Eared doves	O	O^†^	X	X	O	19.7	11.0

X: death; O: survival; ^†^recovered from convulsions; *T*: environmental average temperature during dosing; *P*: photoperiod, in hours of light.

**Table 3:** Mortality in full test.

Individual	1	2	3	4	5	6	7	8	9	10	*T* (°C)	*P*
1st stage												

Dose (mg/kg)	35.4^a^	130.2^d^	479.8^g^	1767.8								
Canaries	O	X	X	X							24.2	12.7

2nd stage												

Dose (mg/kg)	23.2^b^	29.5^b^	37.4^b^	47.5^b^	60.2^c^	76.4^c^	97.0^c^	123.0^e^	156.1^e^	198.0^e^		
Canaries	O*	O*	O*	O*	O	O	O^†^	X	X	X	25.8	12.8
Dose (mg/kg)	769.6	976.5	1065.7	1254.1	1475.7	1736.6	2043.5	2404.8	2829.8	3330.0		
Shiny cowbirds	O*	O*	O	X	O	O	X	X	X	X	15.1	11.0
Eared doves	O*	O*	O	O	O	X	O	X	O	X	19.9	11.3

3rd stage												

Dose (mg/kg)	68.3^c^	68.3^c^	68.3^c^	68.3^c^	68.3^c^	177.2^e^	177.2^e^	177.2^e^	177.2^e^	177.2^e^		
Canaries	O	O	O	O	O	O	O	X	O	O	22.6	12.8
Dose (mg/kg)	985.5	985.5	985.5	985.5	985.5	2558.9	2558.9	2558.9	2558.9	2558.9		
Shiny cowbirds	O	O	O	O	O^†^	O	X	O^†^	X	O	27.4	11.2
Dose (mg/kg)	1451.0	1451.0	1451.0	1451.0	1451.0	3330.0	3330.0	3330.0	3330.0	3330.0		
Eared doves	O	O	O	O	O	X	X	X	X	X	21.5	12.0

3rd stage (2)												

Dose (mg/kg)	105.5^d^	105.5^d^	105.5^d^	105.5^d^	273.9^f^	273.9^f^	273.9^f^	273.9^f^				
Canaries	O	O	O	O	_	X	_	X			19.9	12.9

Dilutions: ^a^0.04, ^b^0.05, ^c^0.1, ^d^0.15, ^e^0.2, ^f^0.4, ^g^0.5; *without clinical signs of intoxication; X: death; O: survival; ^†^recovered from convulsions; *T*: environmental average temperature during dosing; *P*: photoperiod, in hours of light.

**Table 4:** Body weights (±0.05 g for canaries, ±0.1 g for shiny cowbirds and eared doves). Weights are given as predose weight—14-day postdose weight.

Test	Individual	Canaries	Shiny cowbirds	Eared doves
Limit test	1	20.65^a^	53.0–47.4	118.0–128.0
	2	20.35^a^	62.2–54.2	127.5 –126.0
	3	18.45^a^	46.8–39.6	111.8^a^
	4	19.90^a^	49.6^a^	135.7^a^
	5	17.65^a^	62.0^a^	107.2–112.0

1st stage of the full test	1	17.40–21.00		
	2	19.30^a^		
	3	21.15^a^		
	4	17.30^a^		

2nd stage of the full test	1	22.75–21.95	50.0–57.0	131.6–130.0
	2	17.85–19.85	46.0–56.5	105.9–115.0
	3	20.30–23.55	56.1–61.0	101.5–100.0
	4	21.40–24.60	50.1^a^	134.5–122.0
	5	20.15–21.00	57.6–57.8	124.0–122.0
	6	20.55–21.10	48.0–52.8	100.0^a^
	7	18.65–21.10	49.4^a^	143.0–133.3
	8	18.15^a^	42.6^a^	101.0^a^
	9	18.00^a^	65.9^a^	97.0–98.0
	10	19.40^a^	50.3^a^	116.0^a^

3rd stage of full test	1	21.60–22.35	57.2–52.5	124.0–128.0
	2	17.90–23.35	57.0–52.0	107.0–110.0
	3	18.60–21.00	56.2–54.0	122.0-123.0
	4	18.60–22.10	45.0–44.0	88.0–116.0
	5	17.10–18.85	44.7–45.0	107.0-106.0
	6	17.85–18.60	61.5–55.0	108.0^a^
	7	18.25–20.80	49.0^a^	104.0^a^
	8	18.10^a^	49.0–46.0	100.0^a^
	9	20.35–27.35	50.3^a^	116.0^a^
	10	22.10–23.10	52.4–50.0	109.0^a^

3rd stage of the full test (2)	1	19.95–23.35		
	2	22.95–24.60		
	3	20.40–23.00		
	4	23.40–23.60		
	6	20.40^a^		
	8	16.15^a^		

^a^Bird died; only predose weight given.
